# Supplementary figures and images for: The influence of food processing methods on serum parameters, apparent total-tract macronutrient digestibility, fecal microbiota and SCFA content in adult beagles
Source: PLoS One. 2022 Jan 19;17(1):e0262284. doi: 10.1371/journal.pone.0262284 (PMC8769318; doi:10.1371/journal.pone.0262284)

S1 Fig


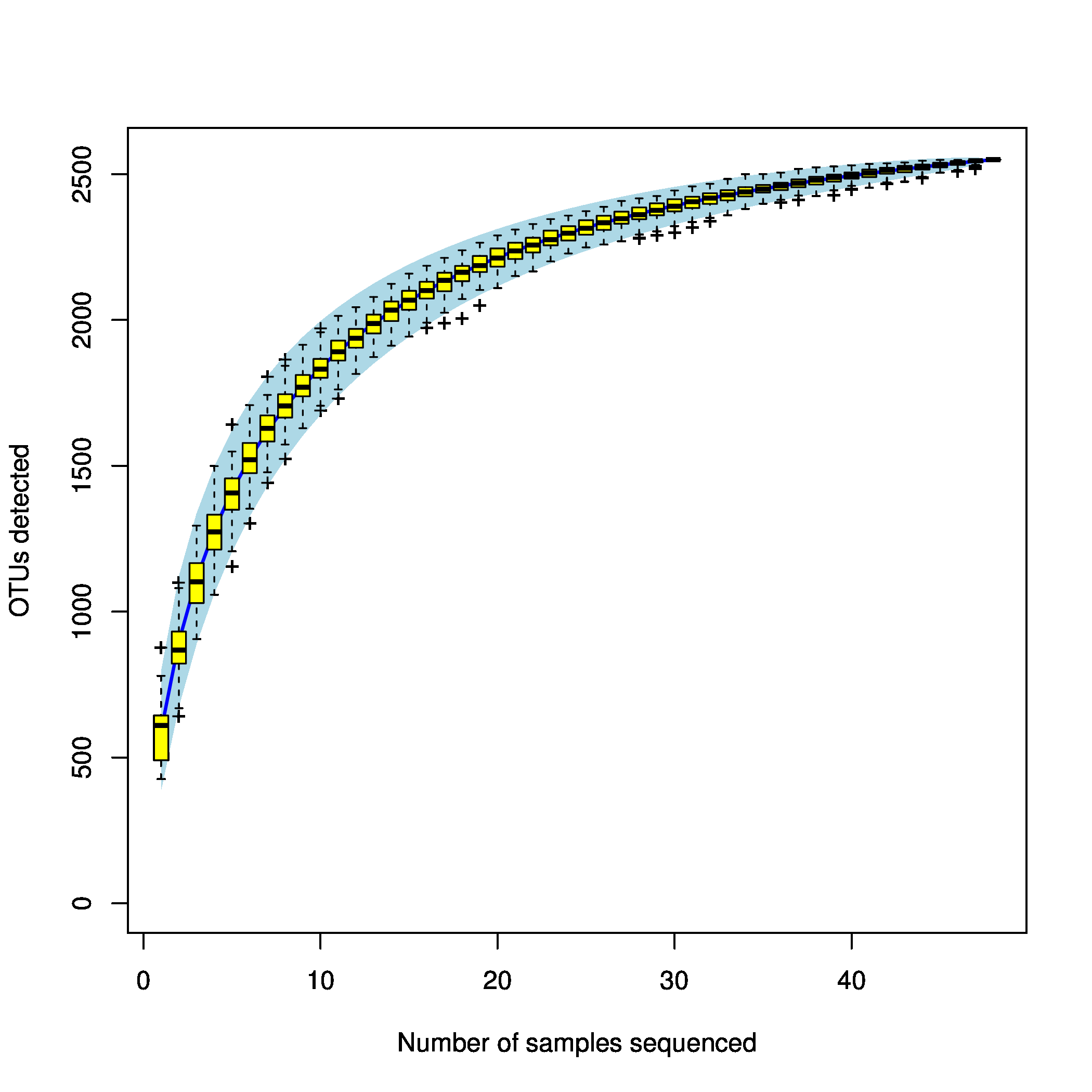


**S1 Fig. OTUs detected in this study.** The curve tends to be flat, indicating adequate sampling.

Supplement: S1 Fig — The curve tends to be flat, indicating adequate sampling. (DOCX) [file pone.0262284.s001.docx]
