# Supplementary material for: The influence of food processing methods on serum parameters, apparent total-tract macronutrient digestibility, fecal microbiota and SCFA content in adult beagles
Source: PLoS One. 2022 Jan 19;17(1):e0262284. doi: 10.1371/journal.pone.0262284 (PMC8769318; doi:10.1371/journal.pone.0262284)
Supplement: S2 Fig — (DOCX) [file pone.0262284.s002.docx]

S2 Fig


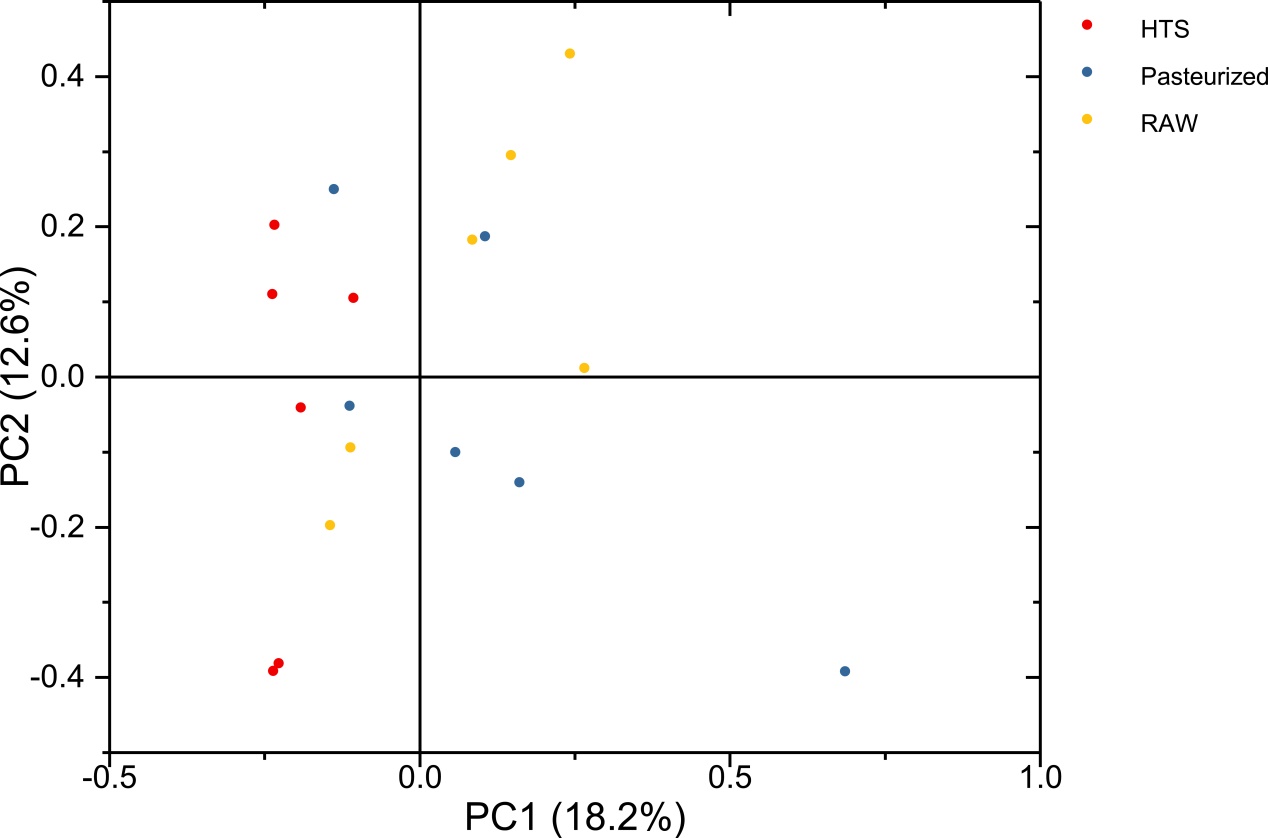


**S2 Fig. Principal component analysis (PCA) plot showing clustering of microbial communities from feces of different processing methods fed dog not diff from each other.**
