# Supplementary material for: The influence of food processing methods on serum parameters, apparent total-tract macronutrient digestibility, fecal microbiota and SCFA content in adult beagles
Source: PLoS One. 2022 Jan 19;17(1):e0262284. doi: 10.1371/journal.pone.0262284 (PMC8769318; doi:10.1371/journal.pone.0262284)
Supplement: S1 Table — (DOCX) [file pone.0262284.s003.docx]

**S1 Table. The blood routine of adult beagles at the beginning (0d).**

| Item | Reference | Raw | Pasteurized | HTS | P-value |
| --- | --- | --- | --- | --- | --- |
| WBC ^†^ (10^9^·L^-1^) | 6.0 - 17.0 | 13.4 ± 0.62 | 13.63 ± 0.91 | 12.55 ± 1.16 | 0.690 |
| Lymph (10^9^·L^-1^) | 0.8 - 5.1 | 3.15 ± 0.39 | 3.32 ± 0.4 | 3.38 ± 0.29 | 0.896 |
| Mon (10^9^·L^-1^) | 0.0 - 1.8 | 0.92 ± 0.06 | 0.93 ± 0.11 | 0.63 ± 0.12 | 0.097 |
| Gran(10^9^·L^-1^) | 4.0 -12.6 | 7.5 ± 0.57 | 6.55 ± 0.54 | 8.2 ± 1.06 | 0.333 |
| RBC (10^12^·L^-1^) | 5.5 - 8.5 | 7.42 ± 0.45 | 7.74 ± 0.29 | 7.81 ± 0.19 | 0.675 |
| HGB (g·L^-1^) | 110 - 190 | 165.5 ± 9.04 | 165 ± 6.89 | 163 ± 8.71 | 0.975 |
| MCV (fL) | 62.0 - 72.0 | 66.7 ± 1.4 | 65.93 ± 1.44 | 65.37 ± 1.75 | 0.829 |
| PLT (10^9^·L^-1^) | 117 - 460 | 312.33 ± 11.12 | 314.5 ± 14.16 | 313.83 ± 19.21 | 0.234 |

^†^WBC, white blood cell count; Lymph, lymph cell count; Mon, monocyte cell count; Gran, neutrophilic granulocyte count; RBC, red blood cell count; HGB, hemoglobin concentration; MCV, mean corpuscular volume; PLT, platelet count
