# Supplementary material for: The influence of food processing methods on serum parameters, apparent total-tract macronutrient digestibility, fecal microbiota and SCFA content in adult beagles
Source: PLoS One. 2022 Jan 19;17(1):e0262284. doi: 10.1371/journal.pone.0262284 (PMC8769318; doi:10.1371/journal.pone.0262284)
Supplement: S2 Table — (DOCX) [file pone.0262284.s004.docx]

**S2 Table. The effect of processing methods on the serum biochemical parameters of adult beagles.**

| Item | Raw | Pasteurized | HTS | P-value |
| --- | --- | --- | --- | --- |
| TP (g·L^-1^) | 65.82±0.73 | 65.27±1.36 | 63.98±1.22 | 0.519 |
| GLB (g·L^-1^) | 37±0.51 | 35.47±0.69 | 35.47±0.95 | 0.267 |
| ALB (g·L^-1^) | 28.82±0.34 | 29.8±1.14 | 28.52±0.55 | 0.472 |
| TC (mM·L^-1^) | 3.88±0.12 | 3.82±0.12 | 3.95±0.11 | 0.731 |
| TG (mM·L^-1^) | 0.79±0.02 | 0.81±0.02 | 0.81±0.02 | 0.704 |
| ALT (U·L^-1^) | 40.1±1.35 | 40.03±1.27 | 41.12±1.93 | 0.858 |
| AST (U·L^-1^) | 33.05±1.16 | 30.9±1.09 | 33.12±1.33 | 0.355 |
| Ca (mM·L^-1^) | 2.55±0.05 | 2.58±0.08 | 2.47±0.08 | 0.533 |
| P (mM·L^-1^) | 1.35±0.05 | 1.47±0.06 | 1.41±0.04 | 0.255 |

^†^TP, total protein; GLB, globulin; ALB, albumin; TC, total cholesterol; TG, triglyceride; ALT, alanine aminotransferase; AST, aspartate aminotransferase; Ca, calcium content; P, phosphorus content.
