# Supplementary material for: The influence of food processing methods on serum parameters, apparent total-tract macronutrient digestibility, fecal microbiota and SCFA content in adult beagles
Source: PLoS One. 2022 Jan 19;17(1):e0262284. doi: 10.1371/journal.pone.0262284 (PMC8769318; doi:10.1371/journal.pone.0262284)
Supplement: S3 Table — (DOCX) [file pone.0262284.s005.docx]

S3 Table. Quality control of 16S rRNA sequencing.

| Item | Raw | Pasteurized | HTS | P-value |
| --- | --- | --- | --- | --- |
| Clean tags | 39679 ± 545 | 39718 ± 167 | 40417 ± 484 | 0.418 |
| Valid tags | 36191 ± 429 | 36218 ± 308 | 37012 ± 468 | 0.299 |
| Valid Length | 414 ± 1.0 | 417 ± 0.7 | 416 ± 1.5 | 0.332 |
| OUT counts | 606 ± 30 | 658 ± 50 | 513 ± 20 | 0.034 |
