# Supplementary material for: The influence of food processing methods on serum parameters, apparent total-tract macronutrient digestibility, fecal microbiota and SCFA content in adult beagles
Source: PLoS One. 2022 Jan 19;17(1):e0262284. doi: 10.1371/journal.pone.0262284 (PMC8769318; doi:10.1371/journal.pone.0262284)
Supplement: S1 File — (DOCX) [file pone.0262284.s006.docx]

S1 File. Raw data of growth performance and apparent total tract digestibility

# PART 1 Feed intake

|  | | No. | JUL 11 | JUL 12 | JUL 13 | JUL 14 | JUL 15 | JUL 16 | JUL 17 |
| --- | --- | --- | --- | --- | --- | --- | --- | --- | --- |
|  |  |  | 1 | 2 | 3 | 4 | 5 | 6 | 7 |
| raw | | 1 | ✓ | ✓ | ✓ | ✓ | ✓ | ✓ | ✓ |
|  |  | 2 | ✓ | ✓ | ✓ | ✓ | ✓ | ✓ | ✓ |
|  |  | 3 | ✓ | ✓ | ✓ | ✓ | ✓ | ✓ | ✓ |
|  |  | 4 | ✓ | ✓ | ✓ | ✓ | ✓ | ✓ | ✓ |
|  |  | 5 | ✓ | ✓ | ✓ | ✓ | ✓ | ✓ | 15 |
|  |  | 6 | ✓ | ✓ | ✓ | ✓ | ✓ | ✓ | ✓ |
|  |  |  |  |  |  |  |  |  |  |
| Pasteurized | | 1 | 50 | 75 | 20 | ✓ | ✓ | ✓ | ✓ |
|  |  | 2 | ✓ | ✓ | ✓ | ✓ | ✓ | ✓ | ✓ |
|  |  | 3 | ✓ | ✓ | ✓ | ✓ | ✓ | ✓ | ✓ |
|  |  | 4 | ✓ | ✓ | ✓ | ✓ | ✓ | ✓ | ✓ |
|  |  | 5 | ✓ | ✓ | ✓ | ✓ | ✓ | ✓ | ✓ |
|  |  | 6 | 80 | 35 | ✓ | ✓ | ✓ | ✓ | ✓ |
|  |  |  |  |  |  |  |  |  |  |
| HTS | | 1 | ✓ | ✓ | ✓ | ✓ | ✓ | ✓ | ✓ |
|  |  | 2 | 20 | 30 | ✓ | ✓ | ✓ | ✓ | ✓ |
|  |  | 3 | ✓ | ✓ | ✓ | ✓ | ✓ | ✓ | ✓ |
|  |  | 4 | ✓ | ✓ | ✓ | ✓ | ✓ | ✓ | ✓ |
|  |  | 5 | ✓ | ✓ | ✓ | ✓ | ✓ | ✓ | ✓ |
|  |  | 6 | ✓ | ✓ | ✓ | ✓ | ✓ | ✓ | ✓ |

| JUL 18 | JUL 19 | JUL 20 | JUL 21 | JUL 22 | JUL 23 | JUL 24 |
| --- | --- | --- | --- | --- | --- | --- |
| 8 | 9 | 10 | 11 | 12 | 13 | 14 |
| ✓ | ✓ | ✓ | ✓ | ✓ | ✓ | ✓ |
| ✓ | ✓ | ✓ | ✓ | ✓ | ✓ | ✓ |
| ✓ | ✓ | ✓ | ✓ | ✓ | ✓ | ✓ |
| ✓ | ✓ | ✓ | ✓ | ✓ | ✓ | ✓ |
| 20 | ✓ | ✓ | ✓ | ✓ | ✓ | ✓ |
| ✓ | ✓ | ✓ | ✓ | ✓ | ✓ | ✓ |
|  |  |  |  |  |  |  |
| ✓ | ✓ | 50 | ✓ | ✓ | ✓ | ✓ |
| ✓ | ✓ | 30 | ✓ | ✓ | ✓ | ✓ |
| ✓ | ✓ | ✓ | ✓ | ✓ | ✓ | ✓ |
| ✓ | ✓ | ✓ | ✓ | ✓ | ✓ | ✓ |
| ✓ | ✓ | ✓ | ✓ | ✓ | ✓ | ✓ |
| ✓ | ✓ | ✓ | ✓ | ✓ | ✓ | ✓ |
|  |  |  |  |  |  |  |
| ✓ | ✓ | ✓ | ✓ | ✓ | ✓ | ✓ |
| ✓ | ✓ | ✓ | ✓ | ✓ | ✓ | ✓ |
| ✓ | ✓ | ✓ | ✓ | ✓ | ✓ | ✓ |
| ✓ | ✓ | ✓ | ✓ | ✓ | ✓ | ✓ |
| ✓ | ✓ | ✓ | ✓ | ✓ | ✓ | ✓ |
| ✓ | ✓ | ✓ | ✓ | ✓ | ✓ | ✓ |

| JUL 25 | JUL 26 | JUL 27 | JUL 28 | JUL 29 | JUL 30 | JUL 31 |
| --- | --- | --- | --- | --- | --- | --- |
| 15 | 16 | 17 | 18 | 19 | 20 | 21 |
| ✓ | ✓ | ✓ | ✓ | ✓ | ✓ | ✓ |
| ✓ | ✓ | ✓ | ✓ | ✓ | ✓ | ✓ |
| ✓ | ✓ | ✓ | ✓ | ✓ | ✓ | ✓ |
| ✓ | ✓ | ✓ | ✓ | ✓ | ✓ | ✓ |
| ✓ | ✓ | ✓ | ✓ | ✓ | ✓ | ✓ |
| ✓ | ✓ | ✓ | ✓ | ✓ | ✓ | ✓ |
|  |  |  |  |  |  |  |
| ✓ | ✓ | ✓ | ✓ | ✓ | ✓ | ✓ |
| ✓ | ✓ | ✓ | ✓ | ✓ | ✓ | ✓ |
| ✓ | ✓ | ✓ | ✓ | ✓ | ✓ | ✓ |
| ✓ | ✓ | ✓ | ✓ | ✓ | ✓ | ✓ |
| ✓ | ✓ | ✓ | ✓ | ✓ | ✓ | ✓ |
| ✓ | ✓ | ✓ | ✓ | ✓ | ✓ | ✓ |
|  |  |  |  |  |  |  |
| ✓ | ✓ | ✓ | ✓ | ✓ | ✓ | ✓ |
| ✓ | ✓ | ✓ | ✓ | ✓ | ✓ | ✓ |
| ✓ | ✓ | ✓ | ✓ | ✓ | ✓ | ✓ |
| ✓ | ✓ | ✓ | ✓ | ✓ | ✓ | ✓ |
| ✓ | ✓ | ✓ | ✓ | ✓ | ✓ | ✓ |
| ✓ | ✓ | ✓ | ✓ | ✓ | ✓ | ✓ |

| AUG 1 | AUG 2 | AUG 3 | AUG 4 | AUG 5 | AUG 6 | AUG 7 |
| --- | --- | --- | --- | --- | --- | --- |
| 22 | 23 | 24 | 25 | 26 | 27 | 28 |
| ✓ | ✓ | ✓ | ✓ | ✓ | ✓ | ✓ |
| ✓ | ✓ | ✓ | ✓ | ✓ | ✓ | ✓ |
| ✓ | ✓ | ✓ | ✓ | ✓ | ✓ | ✓ |
| ✓ | ✓ | ✓ | ✓ | ✓ | ✓ | ✓ |
| ✓ | ✓ | ✓ | ✓ | ✓ | ✓ | ✓ |
| ✓ | ✓ | ✓ | ✓ | ✓ | ✓ | ✓ |
|  |  |  |  |  |  |  |
| ✓ | ✓ | ✓ | ✓ | ✓ | ✓ | ✓ |
| ✓ | ✓ | ✓ | ✓ | ✓ | ✓ | ✓ |
| ✓ | ✓ | ✓ | ✓ | ✓ | ✓ | ✓ |
| ✓ | ✓ | ✓ | ✓ | ✓ | ✓ | ✓ |
| ✓ | ✓ | ✓ | ✓ | ✓ | ✓ | ✓ |
| ✓ | ✓ | ✓ | ✓ | ✓ | ✓ | ✓ |
|  |  |  |  |  |  |  |
| ✓ | ✓ | ✓ | ✓ | ✓ | ✓ | ✓ |
| ✓ | ✓ | ✓ | ✓ | ✓ | ✓ | ✓ |
| ✓ | ✓ | ✓ | ✓ | ✓ | ✓ | ✓ |
| ✓ | ✓ | ✓ | ✓ | ✓ | ✓ | ✓ |
| ✓ | ✓ | ✓ | ✓ | ✓ | ✓ | ✓ |
| ✓ | ✓ | ✓ | ✓ | ✓ | ✓ | ✓ |

| AUG 8 | AUG 9 | AUG 10 | AUG 11 | AUG 12 | AUG 13 | AUG 14 |
| --- | --- | --- | --- | --- | --- | --- |
| 29 | 30 | 31 | 32 | 33 | 34 | 35 |
| ✓ | ✓ | ✓ | ✓ | ✓ | ✓ | ✓ |
| ✓ | ✓ | ✓ | ✓ | ✓ | ✓ | ✓ |
| ✓ | ✓ | ✓ | ✓ | ✓ | ✓ | ✓ |
| ✓ | ✓ | ✓ | ✓ | ✓ | ✓ | ✓ |
| ✓ | ✓ | ✓ | ✓ | ✓ | ✓ | ✓ |
| ✓ | ✓ | ✓ | ✓ | ✓ | ✓ | ✓ |
|  |  |  |  |  |  |  |
| ✓ | ✓ | ✓ | ✓ | ✓ | ✓ | ✓ |
| ✓ | ✓ | ✓ | ✓ | ✓ | ✓ | ✓ |
| ✓ | ✓ | ✓ | ✓ | ✓ | ✓ | ✓ |
| ✓ | ✓ | ✓ | ✓ | ✓ | ✓ | ✓ |
| ✓ | ✓ | ✓ | ✓ | ✓ | ✓ | ✓ |
| ✓ | ✓ | ✓ | ✓ | ✓ | ✓ | ✓ |
|  |  |  |  |  |  |  |
| ✓ | ✓ | ✓ | ✓ | ✓ | ✓ | ✓ |
| ✓ | ✓ | ✓ | ✓ | ✓ | ✓ | ✓ |
| ✓ | ✓ | ✓ | ✓ | ✓ | ✓ | ✓ |
| ✓ | ✓ | ✓ | ✓ | ✓ | ✓ | ✓ |
| ✓ | ✓ | ✓ | ✓ | ✓ | ✓ | ✓ |
| ✓ | ✓ | ✓ | ✓ | ✓ | ✓ | ✓ |

| AUG 15 | AUG 16 | AUG 17 | AUG 18 | AUG 19 | AUG 20 | AUG 21 |
| --- | --- | --- | --- | --- | --- | --- |
| 36 | 37 | 38 | 39 | 40 | 41 | 42 |
| ✓ | ✓ | ✓ | ✓ | ✓ | ✓ | ✓ |
| ✓ | ✓ | ✓ | ✓ | ✓ | ✓ | ✓ |
| ✓ | ✓ | ✓ | ✓ | ✓ | ✓ | ✓ |
| ✓ | ✓ | ✓ | ✓ | ✓ | ✓ | ✓ |
| ✓ | ✓ | ✓ | ✓ | ✓ | ✓ | ✓ |
| ✓ | ✓ | ✓ | ✓ | ✓ | ✓ | ✓ |
|  |  |  |  |  |  |  |
| ✓ | ✓ | ✓ | ✓ | ✓ | ✓ | ✓ |
| ✓ | ✓ | ✓ | ✓ | ✓ | ✓ | ✓ |
| ✓ | ✓ | ✓ | ✓ | ✓ | ✓ | ✓ |
| ✓ | ✓ | ✓ | ✓ | ✓ | ✓ | ✓ |
| ✓ | ✓ | ✓ | ✓ | ✓ | ✓ | ✓ |
| ✓ | ✓ | ✓ | ✓ | ✓ | ✓ | ✓ |
|  |  |  |  |  |  |  |
| ✓ | ✓ | ✓ | ✓ | ✓ | ✓ | ✓ |
| ✓ | ✓ | ✓ | ✓ | ✓ | ✓ | ✓ |
| ✓ | ✓ | ✓ | ✓ | ✓ | ✓ | ✓ |
| ✓ | ✓ | ✓ | ✓ | ✓ | ✓ | ✓ |
| ✓ | ✓ | ✓ | ✓ | ✓ | ✓ | ✓ |
| ✓ | ✓ | ✓ | ✓ | ✓ | ✓ | ✓ |

| AUG 22 | AUG 23 | AUG 24 | AUG 25 | AUG 26 | AUG 27 | AUG 28 |
| --- | --- | --- | --- | --- | --- | --- |
| 43 | 44 | 45 | 46 | 47 | 48 | 49 |
| ✓ | ✓ | ✓ | ✓ | ✓ | ✓ | ✓ |
| ✓ | ✓ | ✓ | ✓ | ✓ | ✓ | ✓ |
| ✓ | ✓ | ✓ | ✓ | ✓ | ✓ | ✓ |
| ✓ | ✓ | ✓ | ✓ | ✓ | ✓ | ✓ |
| ✓ | ✓ | ✓ | ✓ | ✓ | ✓ | ✓ |
| ✓ | ✓ | ✓ | ✓ | ✓ | ✓ | ✓ |
|  |  |  |  |  |  |  |
| ✓ | ✓ | ✓ | ✓ | ✓ | ✓ | ✓ |
| ✓ | ✓ | ✓ | ✓ | ✓ | ✓ | ✓ |
| ✓ | ✓ | ✓ | ✓ | ✓ | ✓ | ✓ |
| ✓ | ✓ | ✓ | ✓ | ✓ | ✓ | ✓ |
| ✓ | ✓ | ✓ | ✓ | ✓ | ✓ | ✓ |
| ✓ | ✓ | ✓ | ✓ | ✓ | ✓ | ✓ |
|  |  |  |  |  |  |  |
| ✓ | ✓ | ✓ | ✓ | ✓ | ✓ | ✓ |
| ✓ | ✓ | ✓ | ✓ | ✓ | ✓ | ✓ |
| ✓ | ✓ | ✓ | ✓ | ✓ | ✓ | ✓ |
| ✓ | ✓ | ✓ | ✓ | ✓ | ✓ | ✓ |
| ✓ | ✓ | ✓ | ✓ | ✓ | ✓ | ✓ |
| ✓ | ✓ | ✓ | ✓ | ✓ | ✓ | ✓ |

| AUG 29 | AUG 30 | AUG 31 | SEP 1 | SEP 2 | SEP 3 | SEP 4 |
| --- | --- | --- | --- | --- | --- | --- |
| 50 | 51 | 52 | 53 | 54 | 55 | 56 |
| ✓ | ✓ | ✓ | ✓ | ✓ | ✓ | ✓ |
| ✓ | ✓ | ✓ | ✓ | ✓ | ✓ | ✓ |
| ✓ | ✓ | ✓ | ✓ | ✓ | ✓ | ✓ |
| ✓ | ✓ | ✓ | ✓ | ✓ | ✓ | ✓ |
| ✓ | ✓ | 50 | 20 | 30 | 20 | ✓ |
| ✓ | ✓ | ✓ | ✓ | ✓ | ✓ | ✓ |
|  |  |  |  |  |  |  |
| ✓ | ✓ | ✓ | ✓ | 95 | ✓ | ✓ |
| ✓ | ✓ | ✓ | ✓ | ✓ | ✓ | ✓ |
| ✓ | ✓ | ✓ | ✓ | ✓ | ✓ | ✓ |
| ✓ | ✓ | ✓ | ✓ | ✓ | ✓ | ✓ |
| ✓ | ✓ | ✓ | ✓ | ✓ | ✓ | ✓ |
| ✓ | ✓ | ✓ | ✓ | ✓ | ✓ | ✓ |
|  |  |  |  |  |  |  |
| ✓ | ✓ | ✓ | ✓ | ✓ | ✓ | ✓ |
| ✓ | ✓ | ✓ | ✓ | ✓ | ✓ | ✓ |
| ✓ | ✓ | ✓ | ✓ | ✓ | ✓ | ✓ |
| ✓ | ✓ | ✓ | ✓ | ✓ | ✓ | ✓ |
| ✓ | ✓ | ✓ | ✓ | ✓ | ✓ | ✓ |
| ✓ | ✓ | ✓ | ✓ | ✓ | ✓ | ✓ |

# PART 2 Body weight

| Group | 56d |
| --- | --- |
| raw | 21.3 |
|  | 20.8 |
|  | 15.6 |
|  | 15.0 |
|  | 12.0 |
|  | 19.5 |
|  | 17.36667 |
| Pasteurized | 17.5 |
|  | 15.0 |
|  | 12.4 |
|  | 14.2 |
|  | 15.3 |
|  | 15.0 |
|  | 14.9 |
| HTS | 11.55 |
|  | 18.6 |
|  | 10.5 |
|  | 18.9 |
|  | 10.1 |
|  | 9.45 |
|  | 13.18333 |

# PART 3 ATTD

| CP |  |  |  |  |  |  |  |  |
| --- | --- | --- | --- | --- | --- | --- | --- | --- |
|  | 1 | 2 | 3 | 4 | 5 | 6 | MEANS | SEM |
| Raw | 89.23 | 90.02 | 88.54 | 87.68 | 91.13 | 88.88 | 89.24667 | 0.491037 |
| Pasteurized | 87.56 | 89.3 | 90.35 | 90.95 | 91.4 | 90.15 | 89.95167 | 0.560865 |
| HTS | 92.85 | 92.22 | 92.56 | 92.15 | 90.6 | 90.18 | 91.76 | 0.448471 |
|  |  |  |  |  |  |  |  |  |
|  |  |  |  |  |  |  |  |  |
| FAT |  |  |  |  |  |  |  |  |
|  | 1 | 2 | 3 | 4 | 5 | 6 | MEANS | SEM |
| Raw | 91.35 | 93.44 | 95.23 | 91.6 | 93.52 | 92.72 | 92.97667 | 0.582939 |
| Pasteurized | 90.46 | 92.01 | 93.54 | 93.12 | 92.65 | 93.89 | 92.61167 | 0.508182 |
| HTS | 93.85 | 92.45 | 91.1 | 95.32 | 92.57 | 94.72 | 93.335 | 0.645171 |
