# Supplementary material for: The influence of food processing methods on serum parameters, apparent total-tract macronutrient digestibility, fecal microbiota and SCFA content in adult beagles
Source: PLoS One. 2022 Jan 19;17(1):e0262284. doi: 10.1371/journal.pone.0262284 (PMC8769318; doi:10.1371/journal.pone.0262284)
Supplement: S2 File — (DOCX) [file pone.0262284.s007.docx]

S2 File. Raw data of blood routine and serum biochemical parameters

For the document:

1: RAW

2: Pasteurized

3: HTS

# PART1. Blood routine

ONEWAY WBC Lymph Mon Gran RBC HGB MCV PLT BY group

/STATISTICS DESCRIPTIVES HOMOGENEITY

/MISSING ANALYSIS

/POSTHOC=DUNCAN LSD ALPHA(0.05).

| Descriptives | | | | | | | |
| --- | --- | --- | --- | --- | --- | --- | --- |
|  | | N | Mean | Std. Deviation | Std. Error | 95% Confidence Interval for Mean | |
|  |  |  |  |  |  | Lower Bound | Upper Bound |
| WBC | 1 | 6 | 12.4167 | .51153 | .20883 | 11.8798 | 12.9535 |
|  | 2 | 6 | 12.3500 | 1.99875 | .81599 | 10.2524 | 14.4476 |
|  | 3 | 6 | 11.7333 | .84538 | .34512 | 10.8462 | 12.6205 |
|  | Total | 18 | 12.1667 | 1.24994 | .29461 | 11.5451 | 12.7882 |
| Lymph | 1 | 6 | 3.3667 | .81158 | .33133 | 2.5150 | 4.2184 |
|  | 2 | 6 | 2.8500 | .64730 | .26426 | 2.1707 | 3.5293 |
|  | 3 | 6 | 3.1000 | .45166 | .18439 | 2.6260 | 3.5740 |
|  | Total | 18 | 3.1056 | .65122 | .15349 | 2.7817 | 3.4294 |
| Mon | 1 | 6 | .9167 | .19408 | .07923 | .7130 | 1.1203 |
|  | 2 | 6 | .8333 | .16330 | .06667 | .6620 | 1.0047 |
|  | 3 | 6 | .7500 | .08367 | .03416 | .6622 | .8378 |
|  | Total | 18 | .8333 | .16088 | .03792 | .7533 | .9133 |
| Gran | 1 | 6 | 10.4833 | 1.20402 | .49154 | 9.2198 | 11.7469 |
|  | 2 | 6 | 10.5500 | 1.67660 | .68447 | 8.7905 | 12.3095 |
|  | 3 | 6 | 8.3833 | 1.41339 | .57701 | 6.9001 | 9.8666 |
|  | Total | 18 | 9.8056 | 1.70655 | .40224 | 8.9569 | 10.6542 |
| RBC | 1 | 6 | 7.3083 | .83994 | .34290 | 6.4269 | 8.1898 |
|  | 2 | 6 | 7.7933 | 1.06795 | .43599 | 6.6726 | 8.9141 |
|  | 3 | 6 | 7.7950 | .65164 | .26603 | 7.1111 | 8.4789 |
|  | Total | 18 | 7.6322 | .85051 | .20047 | 7.2093 | 8.0552 |
| HGB | 1 | 6 | 161.833333 | 17.5888222 | 7.1806066 | 143.374996 | 180.291670 |
|  | 2 | 6 | 159.333333 | 29.2278406 | 11.9322160 | 128.660596 | 190.006071 |
|  | 3 | 6 | 160.833333 | 14.1762713 | 5.7874385 | 145.956249 | 175.710418 |
|  | Total | 18 | 160.666667 | 20.0616696 | 4.7285809 | 150.690233 | 170.643100 |
| MCV | 1 | 6 | 66.3500 | 2.60749 | 1.06450 | 63.6136 | 69.0864 |
|  | 2 | 6 | 67.7667 | 2.83737 | 1.15835 | 64.7890 | 70.7443 |
|  | 3 | 6 | 66.8833 | 1.77247 | .72361 | 65.0232 | 68.7434 |
|  | Total | 18 | 67.0000 | 2.37759 | .56040 | 65.8177 | 68.1823 |
| PLT | 1 | 6 | 349.0000 | 43.52011 | 17.76701 | 303.3284 | 394.6716 |
|  | 2 | 6 | 342.0000 | 32.39753 | 13.22624 | 308.0009 | 375.9991 |
|  | 3 | 6 | 303.3333 | 23.62767 | 9.64595 | 278.5376 | 328.1290 |
|  | Total | 18 | 331.4444 | 38.17024 | 8.99681 | 312.4628 | 350.4261 |

| **Descriptives** | | | |
| --- | --- | --- | --- |
|  | | Minimum | Maximum |
| WBC | 1 | 11.60 | 12.90 |
|  | 2 | 10.10 | 14.80 |
|  | 3 | 10.60 | 12.70 |
|  | Total | 10.10 | 14.80 |
| Lymph | 1 | 2.60 | 4.60 |
|  | 2 | 2.30 | 4.00 |
|  | 3 | 2.50 | 3.70 |
|  | Total | 2.30 | 4.60 |
| Mon | 1 | .70 | 1.20 |
|  | 2 | .60 | 1.00 |
|  | 3 | .60 | .80 |
|  | Total | .60 | 1.20 |
| Gran | 1 | 9.40 | 12.80 |
|  | 2 | 8.00 | 12.30 |
|  | 3 | 6.30 | 10.10 |
|  | Total | 6.30 | 12.80 |
| RBC | 1 | 5.96 | 8.35 |
|  | 2 | 6.31 | 9.19 |
|  | 3 | 6.59 | 8.33 |
|  | Total | 5.96 | 9.19 |
| HGB | 1 | 139.0000 | 183.0000 |
|  | 2 | 123.0000 | 186.0000 |
|  | 3 | 139.0000 | 175.0000 |
|  | Total | 123.0000 | 186.0000 |
| MCV | 1 | 62.70 | 69.30 |
|  | 2 | 64.40 | 71.80 |
|  | 3 | 63.40 | 68.30 |
|  | Total | 62.70 | 71.80 |
| PLT | 1 | 297.00 | 405.00 |
|  | 2 | 305.00 | 393.00 |
|  | 3 | 264.00 | 330.00 |
|  | Total | 264.00 | 405.00 |

| **Test of Homogeneity of Variances** | | | | |
| --- | --- | --- | --- | --- |
|  | Levene Statistic | df1 | df2 | Sig. |
| WBC | 14.229 | 2 | 15 | .000 |
| Lymph | 1.342 | 2 | 15 | .291 |
| Mon | 2.464 | 2 | 15 | .119 |
| Gran | .517 | 2 | 15 | .606 |
| RBC | 1.030 | 2 | 15 | .381 |
| HGB | 2.864 | 2 | 15 | .088 |
| MCV | 1.232 | 2 | 15 | .320 |
| PLT | 2.373 | 2 | 15 | .127 |

| **ANOVA** | | | | | | |
| --- | --- | --- | --- | --- | --- | --- |
|  | | Sum of Squares | df | Mean Square | F | Sig. |
| WBC | Between Groups | 1.703 | 2 | .852 | .514 | .608 |
|  | Within Groups | 24.857 | 15 | 1.657 |  |  |
|  | Total | 26.560 | 17 |  |  |  |
| Lymph | Between Groups | .801 | 2 | .401 | .938 | .413 |
|  | Within Groups | 6.408 | 15 | .427 |  |  |
|  | Total | 7.209 | 17 |  |  |  |
| Mon | Between Groups | .083 | 2 | .042 | 1.752 | .207 |
|  | Within Groups | .357 | 15 | .024 |  |  |
|  | Total | .440 | 17 |  |  |  |
| Gran | Between Groups | 18.218 | 2 | 9.109 | 4.366 | .032 |
|  | Within Groups | 31.292 | 15 | 2.086 |  |  |
|  | Total | 49.509 | 17 |  |  |  |
| RBC | Between Groups | .944 | 2 | .472 | .624 | .549 |
|  | Within Groups | 11.353 | 15 | .757 |  |  |
|  | Total | 12.297 | 17 |  |  |  |
| HGB | Between Groups | 19.000 | 2 | 9.500 | .021 | .979 |
|  | Within Groups | 6823.000 | 15 | 454.867 |  |  |
|  | Total | 6842.000 | 17 |  |  |  |
| MCV | Between Groups | 6.143 | 2 | 3.072 | .512 | .609 |
|  | Within Groups | 89.957 | 15 | 5.997 |  |  |
|  | Total | 96.100 | 17 |  |  |  |
| PLT | Between Groups | 7259.111 | 2 | 3629.556 | 3.109 | .074 |
|  | Within Groups | 17509.333 | 15 | 1167.289 |  |  |
|  | Total | 24768.444 | 17 |  |  |  |

**Post Hoc Tests**

|  | | (I) group | (J) group | Mean差 (I-J) | Std. Error | Sig. | 95% Confidence Interval for Mean | |
| --- | --- | --- | --- | --- | --- | --- | --- | --- |
|  |  |  |  |  |  |  | Lower Bound | Upper Bound |
| WBC | LSD | 1 | 2 | .06667 | .74322 | .930 | -1.5175 | 1.6508 |
|  |  |  | 3 | .68333 | .74322 | .372 | -.9008 | 2.2675 |
|  |  | 2 | 1 | -.06667 | .74322 | .930 | -1.6508 | 1.5175 |
|  |  |  | 3 | .61667 | .74322 | .420 | -.9675 | 2.2008 |
|  |  | 3 | 1 | -.68333 | .74322 | .372 | -2.2675 | .9008 |
|  |  |  | 2 | -.61667 | .74322 | .420 | -2.2008 | .9675 |
| Lymph | LSD | 1 | 2 | .51667 | .37737 | .191 | -.2877 | 1.3210 |
|  |  |  | 3 | .26667 | .37737 | .491 | -.5377 | 1.0710 |
|  |  | 2 | 1 | -.51667 | .37737 | .191 | -1.3210 | .2877 |
|  |  |  | 3 | -.25000 | .37737 | .518 | -1.0543 | .5543 |
|  |  | 3 | 1 | -.26667 | .37737 | .491 | -1.0710 | .5377 |
|  |  |  | 2 | .25000 | .37737 | .518 | -.5543 | 1.0543 |
| Mon | LSD | 1 | 2 | .08333 | .08903 | .364 | -.1064 | .2731 |
|  |  |  | 3 | .16667 | .08903 | .081 | -.0231 | .3564 |
|  |  | 2 | 1 | -.08333 | .08903 | .364 | -.2731 | .1064 |
|  |  |  | 3 | .08333 | .08903 | .364 | -.1064 | .2731 |
|  |  | 3 | 1 | -.16667 | .08903 | .081 | -.3564 | .0231 |
|  |  |  | 2 | -.08333 | .08903 | .364 | -.2731 | .1064 |
| Gran | LSD | 1 | 2 | -.06667 | .83389 | .937 | -1.8441 | 1.7107 |
|  |  |  | 3 | 2.10000^*^ | .83389 | .024 | .3226 | 3.8774 |
|  |  | 2 | 1 | .06667 | .83389 | .937 | -1.7107 | 1.8441 |
|  |  |  | 3 | 2.16667^*^ | .83389 | .020 | .3893 | 3.9441 |
|  |  | 3 | 1 | -2.10000^*^ | .83389 | .024 | -3.8774 | -.3226 |
|  |  |  | 2 | -2.16667^*^ | .83389 | .020 | -3.9441 | -.3893 |
| RBC | LSD | 1 | 2 | -.48500 | .50229 | .350 | -1.5556 | .5856 |
|  |  |  | 3 | -.48667 | .50229 | .348 | -1.5573 | .5839 |
|  |  | 2 | 1 | .48500 | .50229 | .350 | -.5856 | 1.5556 |
|  |  |  | 3 | -.00167 | .50229 | .997 | -1.0723 | 1.0689 |
|  |  | 3 | 1 | .48667 | .50229 | .348 | -.5839 | 1.5573 |
|  |  |  | 2 | .00167 | .50229 | .997 | -1.0689 | 1.0723 |
| HGB | LSD | 1 | 2 | 2.5000000 | 12.3134976 | .842 | -23.745599 | 28.745599 |
|  |  |  | 3 | 1.0000000 | 12.3134976 | .936 | -25.245599 | 27.245599 |
|  |  | 2 | 1 | -2.5000000 | 12.3134976 | .842 | -28.745599 | 23.745599 |
|  |  |  | 3 | -1.5000000 | 12.3134976 | .905 | -27.745599 | 24.745599 |
|  |  | 3 | 1 | -1.0000000 | 12.3134976 | .936 | -27.245599 | 25.245599 |
|  |  |  | 2 | 1.5000000 | 12.3134976 | .905 | -24.745599 | 27.745599 |
| MCV | LSD | 1 | 2 | -1.41667 | 1.41387 | .332 | -4.4303 | 1.5969 |
|  |  |  | 3 | -.53333 | 1.41387 | .711 | -3.5469 | 2.4803 |
|  |  | 2 | 1 | 1.41667 | 1.41387 | .332 | -1.5969 | 4.4303 |
|  |  |  | 3 | .88333 | 1.41387 | .542 | -2.1303 | 3.8969 |
|  |  | 3 | 1 | .53333 | 1.41387 | .711 | -2.4803 | 3.5469 |
|  |  |  | 2 | -.88333 | 1.41387 | .542 | -3.8969 | 2.1303 |
| PLT | LSD | 1 | 2 | 7.00000 | 19.72552 | .728 | -35.0440 | 49.0440 |
|  |  |  | 3 | 45.66667^*^ | 19.72552 | .035 | 3.6227 | 87.7106 |
|  |  | 2 | 1 | -7.00000 | 19.72552 | .728 | -49.0440 | 35.0440 |
|  |  |  | 3 | 38.66667 | 19.72552 | .069 | -3.3773 | 80.7106 |
|  |  | 3 | 1 | -45.66667^*^ | 19.72552 | .035 | -87.7106 | -3.6227 |
|  |  |  | 2 | -38.66667 | 19.72552 | .069 | -80.7106 | 3.3773 |
| *. P < 0.05 | | | | | | | | |

**Homogeneous Subsets**

| **WBC** | | | | | |  |  |
| --- | --- | --- | --- | --- | --- | --- | --- |
|  | group | N | alpha = 0.05 | | |  |  |
|  |  |  | 1 | | |  |  |
| Duncan^a^ | 3 | 6 | 11.7333 | | |  |  |
|  | 2 | 6 | 12.3500 | | |  |  |
|  | 1 | 6 | 12.4167 | | |  |  |
|  | Sig. |  | .397 | | |  |  |
| Means for groups in homogeneous subsets are displayed. | | | | | |  |  |
| Uses Harmonic Mean Sample Size = 6.000. | | | | | |  |  |
| **Lymph** | | | | | |  |  |
|  | group | N | alpha = 0.05 | | |  |  |
|  |  |  | 1 | | |  |  |
| Duncan^a^ | 2 | 6 | 2.8500 | | |  |  |
|  | 3 | 6 | 3.1000 | | |  |  |
|  | 1 | 6 | 3.3667 | | |  |  |
|  | Sig. |  | .213 | | |  |  |
| Means for groups in homogeneous subsets are displayed. | | | | | |  |  |
| Uses Harmonic Mean Sample Size = 6.000. | | | | | |  |  |
| **Mon** | | | | | |  |  |
|  | group | N | alpha = 0.05 | | |  |  |
|  |  |  | 1 | | |  |  |
| Duncan^a^ | 3 | 6 | .7500 | | |  |  |
|  | 2 | 6 | .8333 | | |  |  |
|  | 1 | 6 | .9167 | | |  |  |
|  | Sig. |  | .095 | | |  |  |
| Means for groups in homogeneous subsets are displayed. | | | | | |  |  |
| Uses Harmonic Mean Sample Size = 6.000. | | | | | |  |  |
| **Gran** | | | | | | |  |
|  | group | N | alpha = 0.05 | | | |  |
|  |  |  | 1 | 2 | | |  |
| Duncan^a^ | 3 | 6 | 8.3833 |  | | |  |
|  | 1 | 6 |  | 10.4833 | | |  |
|  | 2 | 6 |  | 10.5500 | | |  |
|  | Sig. |  | 1.000 | .937 | | |  |
| Means for groups in homogeneous subsets are displayed. | | | | | | |  |
| Uses Harmonic Mean Sample Size = 6.000. | | | | | | |  |
| **RBC** | | | | | |  |  |
|  | group | N | alpha = 0.05 | | |  |  |
|  |  |  | 1 | | |  |  |
| Duncan^a^ | 1 | 6 | 7.3083 | | |  |  |
|  | 2 | 6 | 7.7933 | | |  |  |
|  | 3 | 6 | 7.7950 | | |  |  |
|  | Sig. |  | .373 | | |  |  |
| Means for groups in homogeneous subsets are displayed. | | | | | |  |  |
| Uses Harmonic Mean Sample Size = 6.000. | | | | | |  |  |
| **HGB** | | | | | |  |  |
|  | group | N | alpha = 0.05 | | |  |  |
|  |  |  | 1 | | |  |  |
| Duncan^a^ | 2 | 6 | 159.333333 | | |  |  |
|  | 3 | 6 | 160.833333 | | |  |  |
|  | 1 | 6 | 161.833333 | | |  |  |
|  | Sig. |  | .850 | | |  |  |
| Means for groups in homogeneous subsets are displayed. | | | | | |  |  |
| Uses Harmonic Mean Sample Size = 6.000. | | | | | |  |  |
| **MCV** | | | | | |  |  |
|  | group | N | alpha = 0.05 | | |  |  |
|  |  |  | 1 | | |  |  |
| Duncan^a^ | 1 | 6 | 66.3500 | | |  |  |
|  | 3 | 6 | 66.8833 | | |  |  |
|  | 2 | 6 | 67.7667 | | |  |  |
|  | Sig. |  | .357 | | |  |  |
| Means for groups in homogeneous subsets are displayed. | | | | | |  |  |
| Uses Harmonic Mean Sample Size = 6.000. | | | | | |  |  |
| **PLT** | | | | | | | |
|  | group | N | alpha = 0.05 | | | | |
|  |  |  | 1 | | 2 | | |
| Duncan^a^ | 3 | 6 | 303.3333 | |  | | |
|  | 2 | 6 | 342.0000 | | 342.0000 | | |
|  | 1 | 6 |  | | 349.0000 | | |
|  | Sig. |  | .069 | | .728 | | |
| Means for groups in homogeneous subsets are displayed. | | | | | | | |
| Uses Harmonic Mean Sample Size = 6.000. | | | | | | | |

# PART2. Blood routine

ONEWAY TP GLB ALB TC TG ALT AST Ca P BY group

/STATISTICS DESCRIPTIVES HOMOGENEITY

/MISSING ANALYSIS

/POSTHOC=DUNCAN LSD ALPHA(0.05).

| **Descriptives** | | | | | | | |
| --- | --- | --- | --- | --- | --- | --- | --- |
|  | | N | Mean | Std. Deviation | Std. Error | 95% Confidence Interval for Mean | |
|  |  |  |  |  |  | Lower Bound | Upper Bound |
| TP | 1 | 6 | 66.4167 | 4.81432 | 1.96544 | 61.3643 | 71.4690 |
|  | 2 | 6 | 64.6333 | 3.76121 | 1.53551 | 60.6862 | 68.5805 |
|  | 3 | 6 | 64.2500 | 4.21177 | 1.71945 | 59.8300 | 68.6700 |
|  | Total | 18 | 65.1000 | 4.13991 | .97579 | 63.0413 | 67.1587 |
| GLB | 1 | 6 | 37.7500 | 2.65688 | 1.08467 | 34.9618 | 40.5382 |
|  | 2 | 6 | 35.5500 | 1.86091 | .75971 | 33.5971 | 37.5029 |
|  | 3 | 6 | 35.2667 | 4.11323 | 1.67922 | 30.9501 | 39.5832 |
|  | Total | 18 | 36.1889 | 3.06189 | .72169 | 34.6662 | 37.7115 |
| ALB | 1 | 6 | 28.6667 | 4.09276 | 1.67086 | 24.3716 | 32.9618 |
|  | 2 | 6 | 29.0833 | 3.23630 | 1.32122 | 25.6870 | 32.4796 |
|  | 3 | 6 | 28.9833 | 3.30782 | 1.35041 | 25.5120 | 32.4547 |
|  | Total | 18 | 28.9111 | 3.35540 | .79087 | 27.2425 | 30.5797 |
| TC | 1 | 6 | 3.6000 | .57271 | .23381 | 2.9990 | 4.2010 |
|  | 2 | 6 | 4.1667 | 1.09301 | .44622 | 3.0196 | 5.3137 |
|  | 3 | 6 | 3.7500 | .77136 | .31491 | 2.9405 | 4.5595 |
|  | Total | 18 | 3.8389 | .82686 | .19489 | 3.4277 | 4.2501 |
| TG | 1 | 6 | .84167 | .061128 | .024956 | .77752 | .90582 |
|  | 2 | 6 | .87333 | .084063 | .034319 | .78511 | .96155 |
|  | 3 | 6 | .98500 | .093541 | .038188 | .88683 | 1.08317 |
|  | Total | 18 | .90000 | .098757 | .023277 | .85089 | .94911 |
| ALT | 1 | 6 | 39.7333 | 2.93644 | 1.19880 | 36.6517 | 42.8149 |
|  | 2 | 6 | 41.6833 | 2.70438 | 1.10406 | 38.8453 | 44.5214 |
|  | 3 | 6 | 41.1167 | 4.00670 | 1.63573 | 36.9119 | 45.3214 |
|  | Total | 18 | 40.8444 | 3.18105 | .74978 | 39.2625 | 42.4263 |
| AST | 1 | 6 | 31.116667 | 3.6733727 | 1.4996481 | 27.261698 | 34.971635 |
|  | 2 | 6 | 31.016667 | 1.9833473 | .8096982 | 28.935271 | 33.098062 |
|  | 3 | 6 | 32.616667 | 4.2508431 | 1.7353994 | 28.155680 | 37.077653 |
|  | Total | 18 | 31.583333 | 3.3177331 | .7819972 | 29.933463 | 33.233203 |
| Ca | 1 | 6 | 2.2333 | .35455 | .14474 | 1.8613 | 2.6054 |
|  | 2 | 6 | 2.4350 | .53943 | .22022 | 1.8689 | 3.0011 |
|  | 3 | 6 | 2.2283 | .62809 | .25642 | 1.5692 | 2.8875 |
|  | Total | 18 | 2.2989 | .49840 | .11747 | 2.0510 | 2.5467 |
| P | 1 | 6 | 1.1033 | .12226 | .04991 | .9750 | 1.2316 |
|  | 2 | 6 | 1.2500 | .15284 | .06240 | 1.0896 | 1.4104 |
|  | 3 | 6 | 1.1567 | .08824 | .03602 | 1.0641 | 1.2493 |
|  | Total | 18 | 1.1700 | .13209 | .03113 | 1.1043 | 1.2357 |

| **Descriptives** | | | |
| --- | --- | --- | --- |
|  | | Minimum | Maximum |
| TP | 1 | 57.70 | 71.30 |
|  | 2 | 59.80 | 69.20 |
|  | 3 | 59.90 | 71.30 |
|  | Total | 57.70 | 71.30 |
| GLB | 1 | 35.10 | 41.50 |
|  | 2 | 33.90 | 38.40 |
|  | 3 | 30.00 | 41.10 |
|  | Total | 30.00 | 41.50 |
| ALB | 1 | 22.60 | 34.60 |
|  | 2 | 25.90 | 32.80 |
|  | 3 | 23.90 | 32.10 |
|  | Total | 22.60 | 34.60 |
| TC | 1 | 2.80 | 4.30 |
|  | 2 | 2.40 | 5.40 |
|  | 3 | 2.90 | 4.60 |
|  | Total | 2.40 | 5.40 |
| TG | 1 | .760 | .910 |
|  | 2 | .770 | 1.010 |
|  | 3 | .860 | 1.150 |
|  | Total | .760 | 1.150 |
| ALT | 1 | 34.20 | 42.90 |
|  | 2 | 38.50 | 45.70 |
|  | 3 | 37.50 | 47.60 |
|  | Total | 34.20 | 47.60 |
| AST | 1 | 26.0000 | 35.5000 |
|  | 2 | 28.6000 | 33.7000 |
|  | 3 | 25.5000 | 36.0000 |
|  | Total | 25.5000 | 36.0000 |
| Ca | 1 | 1.69 | 2.67 |
|  | 2 | 1.78 | 3.09 |
|  | 3 | 1.47 | 3.15 |
|  | Total | 1.47 | 3.15 |
| P | 1 | .92 | 1.22 |
|  | 2 | 1.06 | 1.43 |
|  | 3 | 1.09 | 1.32 |
|  | Total | .92 | 1.43 |

| **Test of Homogeneity of Variances** | | | | |
| --- | --- | --- | --- | --- |
|  | Levene Statistic | df1 | df2 | Sig. |
| TP | .021 | 2 | 15 | .979 |
| GLB | 1.428 | 2 | 15 | .271 |
| ALB | .099 | 2 | 15 | .907 |
| TC | 1.829 | 2 | 15 | .195 |
| TG | .080 | 2 | 15 | .923 |
| ALT | 1.112 | 2 | 15 | .355 |
| AST | 2.043 | 2 | 15 | .164 |
| Ca | 1.622 | 2 | 15 | .230 |
| P | 2.470 | 2 | 15 | .118 |

| **ANOVA** | | | | | | |
| --- | --- | --- | --- | --- | --- | --- |
|  | | Sum of Squares | df | Mean Square | F | Sig. |
| TP | Between Groups | 16.043 | 2 | 8.022 | .437 | .654 |
|  | Within Groups | 275.317 | 15 | 18.354 |  |  |
|  | Total | 291.360 | 17 |  |  |  |
| GLB | Between Groups | 22.174 | 2 | 11.087 | 1.212 | .325 |
|  | Within Groups | 137.203 | 15 | 9.147 |  |  |
|  | Total | 159.378 | 17 |  |  |  |
| ALB | Between Groups | .568 | 2 | .284 | .022 | .978 |
|  | Within Groups | 190.830 | 15 | 12.722 |  |  |
|  | Total | 191.398 | 17 |  |  |  |
| TC | Between Groups | 1.034 | 2 | .517 | .733 | .497 |
|  | Within Groups | 10.588 | 15 | .706 |  |  |
|  | Total | 11.623 | 17 |  |  |  |
| TG | Between Groups | .068 | 2 | .034 | 5.219 | .019 |
|  | Within Groups | .098 | 15 | .007 |  |  |
|  | Total | .166 | 17 |  |  |  |
| ALT | Between Groups | 12.074 | 2 | 6.037 | .566 | .579 |
|  | Within Groups | 159.950 | 15 | 10.663 |  |  |
|  | Total | 172.024 | 17 |  |  |  |
| AST | Between Groups | 9.640 | 2 | 4.820 | .407 | .673 |
|  | Within Groups | 177.485 | 15 | 11.832 |  |  |
|  | Total | 187.125 | 17 |  |  |  |
| Ca | Between Groups | .167 | 2 | .083 | .308 | .739 |
|  | Within Groups | 4.056 | 15 | .270 |  |  |
|  | Total | 4.223 | 17 |  |  |  |
| P | Between Groups | .066 | 2 | .033 | 2.152 | .151 |
|  | Within Groups | .230 | 15 | .015 |  |  |
|  | Total | .297 | 17 |  |  |  |

**Post Hoc Tests**

| 因变量 | | (I) group | (J) group | Mean差 (I-J) | Std. Error | Sig. | 95% Confidence Interval for Mean | |
| --- | --- | --- | --- | --- | --- | --- | --- | --- |
|  |  |  |  |  |  |  | Lower Bound | Upper Bound |
| TP | LSD | 1 | 2 | 1.78333 | 2.47349 | .482 | -3.4888 | 7.0555 |
|  |  |  | 3 | 2.16667 | 2.47349 | .395 | -3.1055 | 7.4388 |
|  |  | 2 | 1 | -1.78333 | 2.47349 | .482 | -7.0555 | 3.4888 |
|  |  |  | 3 | .38333 | 2.47349 | .879 | -4.8888 | 5.6555 |
|  |  | 3 | 1 | -2.16667 | 2.47349 | .395 | -7.4388 | 3.1055 |
|  |  |  | 2 | -.38333 | 2.47349 | .879 | -5.6555 | 4.8888 |
| GLB | LSD | 1 | 2 | 2.20000 | 1.74613 | .227 | -1.5218 | 5.9218 |
|  |  |  | 3 | 2.48333 | 1.74613 | .175 | -1.2385 | 6.2051 |
|  |  | 2 | 1 | -2.20000 | 1.74613 | .227 | -5.9218 | 1.5218 |
|  |  |  | 3 | .28333 | 1.74613 | .873 | -3.4385 | 4.0051 |
|  |  | 3 | 1 | -2.48333 | 1.74613 | .175 | -6.2051 | 1.2385 |
|  |  |  | 2 | -.28333 | 1.74613 | .873 | -4.0051 | 3.4385 |
| ALB | LSD | 1 | 2 | -.41667 | 2.05929 | .842 | -4.8059 | 3.9726 |
|  |  |  | 3 | -.31667 | 2.05929 | .880 | -4.7059 | 4.0726 |
|  |  | 2 | 1 | .41667 | 2.05929 | .842 | -3.9726 | 4.8059 |
|  |  |  | 3 | .10000 | 2.05929 | .962 | -4.2893 | 4.4893 |
|  |  | 3 | 1 | .31667 | 2.05929 | .880 | -4.0726 | 4.7059 |
|  |  |  | 2 | -.10000 | 2.05929 | .962 | -4.4893 | 4.2893 |
| TC | LSD | 1 | 2 | -.56667 | .48507 | .261 | -1.6006 | .4672 |
|  |  |  | 3 | -.15000 | .48507 | .761 | -1.1839 | .8839 |
|  |  | 2 | 1 | .56667 | .48507 | .261 | -.4672 | 1.6006 |
|  |  |  | 3 | .41667 | .48507 | .404 | -.6172 | 1.4506 |
|  |  | 3 | 1 | .15000 | .48507 | .761 | -.8839 | 1.1839 |
|  |  |  | 2 | -.41667 | .48507 | .404 | -1.4506 | .6172 |
| TG | LSD | 1 | 2 | -.031667 | .046611 | .507 | -.13102 | .06768 |
|  |  |  | 3 | -.143333^*^ | .046611 | .008 | -.24268 | -.04398 |
|  |  | 2 | 1 | .031667 | .046611 | .507 | -.06768 | .13102 |
|  |  |  | 3 | -.111667^*^ | .046611 | .030 | -.21102 | -.01232 |
|  |  | 3 | 1 | .143333^*^ | .046611 | .008 | .04398 | .24268 |
|  |  |  | 2 | .111667^*^ | .046611 | .030 | .01232 | .21102 |
| ALT | LSD | 1 | 2 | -1.95000 | 1.88532 | .317 | -5.9685 | 2.0685 |
|  |  |  | 3 | -1.38333 | 1.88532 | .474 | -5.4018 | 2.6351 |
|  |  | 2 | 1 | 1.95000 | 1.88532 | .317 | -2.0685 | 5.9685 |
|  |  |  | 3 | .56667 | 1.88532 | .768 | -3.4518 | 4.5851 |
|  |  | 3 | 1 | 1.38333 | 1.88532 | .474 | -2.6351 | 5.4018 |
|  |  |  | 2 | -.56667 | 1.88532 | .768 | -4.5851 | 3.4518 |
| AST | LSD | 1 | 2 | .1000000 | 1.9859786 | .961 | -4.133013 | 4.333013 |
|  |  |  | 3 | -1.5000000 | 1.9859786 | .462 | -5.733013 | 2.733013 |
|  |  | 2 | 1 | -.1000000 | 1.9859786 | .961 | -4.333013 | 4.133013 |
|  |  |  | 3 | -1.6000000 | 1.9859786 | .433 | -5.833013 | 2.633013 |
|  |  | 3 | 1 | 1.5000000 | 1.9859786 | .462 | -2.733013 | 5.733013 |
|  |  |  | 2 | 1.6000000 | 1.9859786 | .433 | -2.633013 | 5.833013 |
| Ca | LSD | 1 | 2 | -.20167 | .30022 | .512 | -.8416 | .4382 |
|  |  |  | 3 | .00500 | .30022 | .987 | -.6349 | .6449 |
|  |  | 2 | 1 | .20167 | .30022 | .512 | -.4382 | .8416 |
|  |  |  | 3 | .20667 | .30022 | .502 | -.4332 | .8466 |
|  |  | 3 | 1 | -.00500 | .30022 | .987 | -.6449 | .6349 |
|  |  |  | 2 | -.20667 | .30022 | .502 | -.8466 | .4332 |
| P | LSD | 1 | 2 | -.14667 | .07156 | .058 | -.2992 | .0059 |
|  |  |  | 3 | -.05333 | .07156 | .468 | -.2059 | .0992 |
|  |  | 2 | 1 | .14667 | .07156 | .058 | -.0059 | .2992 |
|  |  |  | 3 | .09333 | .07156 | .212 | -.0592 | .2459 |
|  |  | 3 | 1 | .05333 | .07156 | .468 | -.0992 | .2059 |
|  |  |  | 2 | -.09333 | .07156 | .212 | -.2459 | .0592 |
| *. P < 0.05 | | | | | | | | |

**Homogeneous Subsets**

| **TP** | | | | | | | | |  |
| --- | --- | --- | --- | --- | --- | --- | --- | --- | --- |
|  | | group | | N | | alpha = 0.05 | | |  |
|  | |  |  |  |  | 1 | | |  |
| Duncan^a^ | | 3 | | 6 | | 64.2500 | | |  |
|  |  | 2 | | 6 | | 64.6333 | | |  |
|  |  | 1 | | 6 | | 66.4167 | | |  |
|  |  | Sig. | |  | | .420 | | |  |
| Means for groups in homogeneous subsets are displayed. | | | | | | | | |  |
| Uses Harmonic Mean Sample Size = 6.000. | | | | | | | | |  |
| **GLB** | | | | | | | | |  |
|  | | group | | N | | alpha = 0.05 | | |  |
|  | |  |  |  |  | 1 | | |  |
| Duncan^a^ | | 3 | | 6 | | 35.2667 | | |  |
|  |  | 2 | | 6 | | 35.5500 | | |  |
|  |  | 1 | | 6 | | 37.7500 | | |  |
|  |  | Sig. | |  | | .197 | | |  |
| Means for groups in homogeneous subsets are displayed. | | | | | | | | |  |
| Uses Harmonic Mean Sample Size = 6.000. | | | | | | | | |  |
| **ALB** | | | | | | | | |  |
|  | | group | | N | | alpha = 0.05 | | |  |
|  | |  |  |  |  | 1 | | |  |
| Duncan^a^ | | 1 | | 6 | | 28.6667 | | |  |
|  |  | 3 | | 6 | | 28.9833 | | |  |
|  |  | 2 | | 6 | | 29.0833 | | |  |
|  |  | Sig. | |  | | .851 | | |  |
| Means for groups in homogeneous subsets are displayed. | | | | | | | | |  |
| Uses Harmonic Mean Sample Size = 6.000. | | | | | | | | |  |
| **TC** | | | | | | | | |  |
|  | | group | | N | | alpha = 0.05 | | |  |
|  | |  |  |  |  | 1 | | |  |
| Duncan^a^ | | 1 | | 6 | | 3.6000 | | |  |
|  |  | 3 | | 6 | | 3.7500 | | |  |
|  |  | 2 | | 6 | | 4.1667 | | |  |
|  |  | Sig. | |  | | .285 | | |  |
| Means for groups in homogeneous subsets are displayed. | | | | | | | | |  |
| Uses Harmonic Mean Sample Size = 6.000. | | | | | | | | |  |
| **TG** | | | | | | | | | |
|  | | group | | N | | alpha = 0.05 | | | |
|  | |  |  |  |  | 1 | 2 | | |
| Duncan^a^ | | 1 | | 6 | | .84167 |  | | |
|  |  | 2 | | 6 | | .87333 |  | | |
|  |  | 3 | | 6 | |  | .98500 | | |
|  |  | Sig. | |  | | .507 | 1.000 | | |
| Means for groups in homogeneous subsets are displayed. | | | | | | | | | |
| Uses Harmonic Mean Sample Size = 6.000. | | | | | | | | | |
| **ALT** | | | | | | | |  |  |
|  | group | | N | | alpha = 0.05 | | |  |  |
|  |  |  |  |  | 1 | | |  |  |
| Duncan^a^ | 1 | | 6 | | 39.7333 | | |  |  |
|  | 3 | | 6 | | 41.1167 | | |  |  |
|  | 2 | | 6 | | 41.6833 | | |  |  |
|  | Sig. | |  | | .342 | | |  |  |
| Means for groups in homogeneous subsets are displayed. | | | | | | | |  |  |
| Uses Harmonic Mean Sample Size = 6.000. | | | | | | | |  |  |
| **AST** | | | | | | | |  |  |
|  | group | | N | | alpha = 0.05 | | |  |  |
|  |  |  |  |  | 1 | | |  |  |
| Duncan^a^ | 2 | | 6 | | 31.016667 | | |  |  |
|  | 1 | | 6 | | 31.116667 | | |  |  |
|  | 3 | | 6 | | 32.616667 | | |  |  |
|  | Sig. | |  | | .457 | | |  |  |
| Means for groups in homogeneous subsets are displayed. | | | | | | | |  |  |
| Uses Harmonic Mean Sample Size = 6.000. | | | | | | | |  |  |
| **Ca** | | | | | | | |  |  |
|  | group | | N | | alpha = 0.05 | | |  |  |
|  |  |  |  |  | 1 | | |  |  |
| Duncan^a^ | 3 | | 6 | | 2.2283 | | |  |  |
|  | 1 | | 6 | | 2.2333 | | |  |  |
|  | 2 | | 6 | | 2.4350 | | |  |  |
|  | Sig. | |  | | .524 | | |  |  |
| Means for groups in homogeneous subsets are displayed. | | | | | | | |  |  |
| Uses Harmonic Mean Sample Size = 6.000. | | | | | | | |  |  |
| **P** | | | | | | | |  |  |
|  | group | | N | | alpha = 0.05 | | |  |  |
|  |  |  |  |  | 1 | | |  |  |
| Duncan^a^ | 1 | | 6 | | 1.1033 | | |  |  |
|  | 3 | | 6 | | 1.1567 | | |  |  |
|  | 2 | | 6 | | 1.2500 | | |  |  |
|  | Sig. | |  | | .070 | | |  |  |
| Means for groups in homogeneous subsets are displayed. | | | | | | | |  |  |
| Uses Harmonic Mean Sample Size = 6.000. | | | | | | | |  |  |
