# Supplementary material for: The influence of food processing methods on serum parameters, apparent total-tract macronutrient digestibility, fecal microbiota and SCFA content in adult beagles
Source: PLoS One. 2022 Jan 19;17(1):e0262284. doi: 10.1371/journal.pone.0262284 (PMC8769318; doi:10.1371/journal.pone.0262284)
Supplement: S3 File — (DOCX) [file pone.0262284.s008.docx]

S3 File. Raw data of SCFA

For the document:

1: RAW

2: Pasteurized

3: HTS

ONEWAY Acetic Propionic Butyric Isovaleric Valeric Total BY GROUP

/STATISTICS DESCRIPTIVES HOMOGENEITY

/MISSING ANALYSIS

/POSTHOC=DUNCAN LSD T3 ALPHA(0.05).

| **Descriptives** | | | | | | | |
| --- | --- | --- | --- | --- | --- | --- | --- |
|  | | N | Mean | Std. Deviation | Std. Error | 95% Confidence Interval for Mean | |
|  |  |  |  |  |  | Lower Bound | Upper Bound |
| Acetic | 1 | 6 | 3.53667 | .906922 | .370249 | 2.58491 | 4.48842 |
|  | 2 | 6 | 5.21667 | .695116 | .283780 | 4.48719 | 5.94615 |
|  | 3 | 6 | 4.90833 | .605885 | .247352 | 4.27250 | 5.54417 |
|  | Total | 18 | 4.55389 | 1.027900 | .242278 | 4.04273 | 5.06505 |
| Propionic | 1 | 6 | 3.58833 | .648519 | .264757 | 2.90775 | 4.26891 |
|  | 2 | 6 | 4.37500 | .709049 | .289468 | 3.63090 | 5.11910 |
|  | 3 | 6 | 4.59667 | .407120 | .166206 | 4.16942 | 5.02391 |
|  | Total | 18 | 4.18667 | .720082 | .169725 | 3.82858 | 4.54475 |
| Butyric | 1 | 6 | .78833 | .240617 | .098232 | .53582 | 1.04085 |
|  | 2 | 6 | 1.35167 | .483298 | .197305 | .84448 | 1.85886 |
|  | 3 | 6 | 1.52833 | .383532 | .156576 | 1.12584 | 1.93083 |
|  | Total | 18 | 1.22278 | .484171 | .114120 | .98201 | 1.46355 |
| Isovaleric | 1 | 6 | .21500 | .093755 | .038275 | .11661 | .31339 |
|  | 2 | 6 | .24833 | .082077 | .033508 | .16220 | .33447 |
|  | 3 | 6 | .30667 | .058878 | .024037 | .24488 | .36846 |
|  | Total | 18 | .25667 | .084296 | .019869 | .21475 | .29859 |
| Valeric | 1 | 6 | .26500 | .226164 | .092331 | .02766 | .50234 |
|  | 2 | 6 | .27667 | .200865 | .082003 | .06587 | .48746 |
|  | 3 | 6 | .04333 | .042740 | .017448 | -.00152 | .08819 |
|  | Total | 18 | .19500 | .199123 | .046934 | .09598 | .29402 |
| Total | 1 | 6 | 8.39333 | 1.739490 | .710144 | 6.56785 | 10.21882 |
|  | 2 | 6 | 11.46833 | 1.693191 | .691242 | 9.69144 | 13.24523 |
|  | 3 | 6 | 11.38333 | 1.130481 | .461517 | 10.19697 | 12.56970 |
|  | Total | 18 | 10.41500 | 2.067386 | .487288 | 9.38691 | 11.44309 |

| **Descriptives** | | | |
| --- | --- | --- | --- |
|  | | Minimum | Maximum |
| Acetic | 1 | 2.580 | 5.110 |
|  | 2 | 4.120 | 6.050 |
|  | 3 | 3.900 | 5.610 |
|  | Total | 2.580 | 6.050 |
| Propionic | 1 | 2.350 | 4.150 |
|  | 2 | 3.090 | 5.110 |
|  | 3 | 4.030 | 5.120 |
|  | Total | 2.350 | 5.120 |
| Butyric | 1 | .460 | 1.050 |
|  | 2 | .820 | 1.980 |
|  | 3 | .850 | 1.900 |
|  | Total | .460 | 1.980 |
| Isovaleric | 1 | .110 | .390 |
|  | 2 | .160 | .390 |
|  | 3 | .250 | .410 |
|  | Total | .110 | .410 |
| Valeric | 1 | .080 | .690 |
|  | 2 | .000 | .520 |
|  | 3 | .000 | .100 |
|  | Total | .000 | .690 |
| Total | 1 | 5.700 | 10.820 |
|  | 2 | 9.420 | 13.770 |
|  | 3 | 9.660 | 12.220 |
|  | Total | 5.700 | 13.770 |

| **Test of Homogeneity of Variances** | | | | |
| --- | --- | --- | --- | --- |
|  | Levene Statistic | df1 | df2 | Sig. |
| Acetic | .508 | 2 | 15 | .612 |
| Propionic | .290 | 2 | 15 | .752 |
| Butyric | 1.397 | 2 | 15 | .278 |
| Isovaleric | .291 | 2 | 15 | .752 |
| Valeric | 3.015 | 2 | 15 | .079 |
| Total | .315 | 2 | 15 | .734 |

| **ANOVA** | | | | | | |
| --- | --- | --- | --- | --- | --- | --- |
|  | | Sum of Squares | df | Mean Square | F | Sig. |
| Acetic | Between Groups | 9.598 | 2 | 4.799 | 8.606 | .003 |
|  | Within Groups | 8.364 | 15 | .558 |  |  |
|  | Total | 17.962 | 17 |  |  |  |
| Propionic | Between Groups | 3.369 | 2 | 1.685 | 4.641 | .027 |
|  | Within Groups | 5.445 | 15 | .363 |  |  |
|  | Total | 8.815 | 17 |  |  |  |
| Butyric | Between Groups | 1.792 | 2 | .896 | 6.130 | .011 |
|  | Within Groups | 2.193 | 15 | .146 |  |  |
|  | Total | 3.985 | 17 |  |  |  |
| Isovaleric | Between Groups | .026 | 2 | .013 | 2.040 | .165 |
|  | Within Groups | .095 | 15 | .006 |  |  |
|  | Total | .121 | 17 |  |  |  |
| Valeric | Between Groups | .207 | 2 | .104 | 3.334 | .063 |
|  | Within Groups | .467 | 15 | .031 |  |  |
|  | Total | .674 | 17 |  |  |  |
| Total | Between Groups | 36.806 | 2 | 18.403 | 7.699 | .005 |
|  | Within Groups | 35.854 | 15 | 2.390 |  |  |
|  | Total | 72.659 | 17 |  |  |  |

|  | | | | | | | | |
| --- | --- | --- | --- | --- | --- | --- | --- | --- |
|  | | (I) GROUP | (J) GROUP | Mean (I-J) | Std. Error | Sig. | 95% Confidence Interval for Mean | |
|  |  |  |  |  |  |  | Lower Bound | Upper Bound |
| Acetic | LSD | 1 | 2 | -1.680000^*^ | .431121 | .001 | -2.59891 | -.76109 |
|  |  |  | 3 | -1.371667^*^ | .431121 | .006 | -2.29058 | -.45275 |
|  |  | 2 | 1 | 1.680000^*^ | .431121 | .001 | .76109 | 2.59891 |
|  |  |  | 3 | .308333 | .431121 | .485 | -.61058 | 1.22725 |
|  |  | 3 | 1 | 1.371667^*^ | .431121 | .006 | .45275 | 2.29058 |
|  |  |  | 2 | -.308333 | .431121 | .485 | -1.22725 | .61058 |
|  | Dunnett T3 | 1 | 2 | -1.680000^*^ | .466493 | .015 | -3.01554 | -.34446 |
|  |  |  | 3 | -1.371667^*^ | .445272 | .038 | -2.66454 | -.07879 |
|  |  | 2 | 1 | 1.680000^*^ | .466493 | .015 | .34446 | 3.01554 |
|  |  |  | 3 | .308333 | .376449 | .799 | -.76011 | 1.37677 |
|  |  | 3 | 1 | 1.371667^*^ | .445272 | .038 | .07879 | 2.66454 |
|  |  |  | 2 | -.308333 | .376449 | .799 | -1.37677 | .76011 |
| Propionic | LSD | 1 | 2 | -.786667^*^ | .347862 | .039 | -1.52812 | -.04522 |
|  |  |  | 3 | -1.008333^*^ | .347862 | .011 | -1.74978 | -.26688 |
|  |  | 2 | 1 | .786667^*^ | .347862 | .039 | .04522 | 1.52812 |
|  |  |  | 3 | -.221667 | .347862 | .534 | -.96312 | .51978 |
|  |  | 3 | 1 | 1.008333^*^ | .347862 | .011 | .26688 | 1.74978 |
|  |  |  | 2 | .221667 | .347862 | .534 | -.51978 | .96312 |
|  | Dunnett T3 | 1 | 2 | -.786667 | .392285 | .191 | -1.89794 | .32461 |
|  |  |  | 3 | -1.008333^*^ | .312603 | .032 | -1.92291 | -.09376 |
|  |  | 2 | 1 | .786667 | .392285 | .191 | -.32461 | 1.89794 |
|  |  |  | 3 | -.221667 | .333791 | .878 | -1.20976 | .76643 |
|  |  | 3 | 1 | 1.008333^*^ | .312603 | .032 | .09376 | 1.92291 |
|  |  |  | 2 | .221667 | .333791 | .878 | -.76643 | 1.20976 |
| Butyric | LSD | 1 | 2 | -.563333^*^ | .220749 | .022 | -1.03385 | -.09282 |
|  |  |  | 3 | -.740000^*^ | .220749 | .004 | -1.21051 | -.26949 |
|  |  | 2 | 1 | .563333^*^ | .220749 | .022 | .09282 | 1.03385 |
|  |  |  | 3 | -.176667 | .220749 | .436 | -.64718 | .29385 |
|  |  | 3 | 1 | .740000^*^ | .220749 | .004 | .26949 | 1.21051 |
|  |  |  | 2 | .176667 | .220749 | .436 | -.29385 | .64718 |
|  | Dunnett T3 | 1 | 2 | -.563333 | .220406 | .097 | -1.22881 | .10214 |
|  |  |  | 3 | -.740000^*^ | .184839 | .010 | -1.28082 | -.19918 |
|  |  | 2 | 1 | .563333 | .220406 | .097 | -.10214 | 1.22881 |
|  |  |  | 3 | -.176667 | .251884 | .860 | -.89576 | .54242 |
|  |  | 3 | 1 | .740000^*^ | .184839 | .010 | .19918 | 1.28082 |
|  |  |  | 2 | .176667 | .251884 | .860 | -.54242 | .89576 |
| Isovaleric | LSD | 1 | 2 | -.033333 | .045939 | .479 | -.13125 | .06458 |
|  |  |  | 3 | -.091667 | .045939 | .064 | -.18958 | .00625 |
|  |  | 2 | 1 | .033333 | .045939 | .479 | -.06458 | .13125 |
|  |  |  | 3 | -.058333 | .045939 | .223 | -.15625 | .03958 |
|  |  | 3 | 1 | .091667 | .045939 | .064 | -.00625 | .18958 |
|  |  |  | 2 | .058333 | .045939 | .223 | -.03958 | .15625 |
|  | Dunnett T3 | 1 | 2 | -.033333 | .050870 | .882 | -.17768 | .11102 |
|  |  |  | 3 | -.091667 | .045197 | .195 | -.22389 | .04056 |
|  |  | 2 | 1 | .033333 | .050870 | .882 | -.11102 | .17768 |
|  |  |  | 3 | -.058333 | .041238 | .444 | -.17714 | .06047 |
|  |  | 3 | 1 | .091667 | .045197 | .195 | -.04056 | .22389 |
|  |  |  | 2 | .058333 | .041238 | .444 | -.06047 | .17714 |
| Valeric | LSD | 1 | 2 | -.011667 | .101830 | .910 | -.22871 | .20538 |
|  |  |  | 3 | .221667^*^ | .101830 | .046 | .00462 | .43871 |
|  |  | 2 | 1 | .011667 | .101830 | .910 | -.20538 | .22871 |
|  |  |  | 3 | .233333^*^ | .101830 | .037 | .01629 | .45038 |
|  |  | 3 | 1 | -.221667^*^ | .101830 | .046 | -.43871 | -.00462 |
|  |  |  | 2 | -.233333^*^ | .101830 | .037 | -.45038 | -.01629 |
|  | Dunnett T3 | 1 | 2 | -.011667 | .123489 | 1.000 | -.36186 | .33853 |
|  |  |  | 3 | .221667 | .093965 | .154 | -.08979 | .53312 |
|  |  | 2 | 1 | .011667 | .123489 | 1.000 | -.33853 | .36186 |
|  |  |  | 3 | .233333 | .083838 | .091 | -.04285 | .50952 |
|  |  | 3 | 1 | -.221667 | .093965 | .154 | -.53312 | .08979 |
|  |  |  | 2 | -.233333 | .083838 | .091 | -.50952 | .04285 |
| Total | LSD | 1 | 2 | -3.075000^*^ | .892606 | .004 | -4.97754 | -1.17246 |
|  |  |  | 3 | -2.990000^*^ | .892606 | .004 | -4.89254 | -1.08746 |
|  |  | 2 | 1 | 3.075000^*^ | .892606 | .004 | 1.17246 | 4.97754 |
|  |  |  | 3 | .085000 | .892606 | .925 | -1.81754 | 1.98754 |
|  |  | 3 | 1 | 2.990000^*^ | .892606 | .004 | 1.08746 | 4.89254 |
|  |  |  | 2 | -.085000 | .892606 | .925 | -1.98754 | 1.81754 |
|  | Dunnett T3 | 1 | 2 | -3.075000^*^ | .991020 | .032 | -5.87881 | -.27119 |
|  |  |  | 3 | -2.990000^*^ | .846937 | .019 | -5.45723 | -.52277 |
|  |  | 2 | 1 | 3.075000^*^ | .991020 | .032 | .27119 | 5.87881 |
|  |  |  | 3 | .085000 | .831152 | .999 | -2.32847 | 2.49847 |
|  |  | 3 | 1 | 2.990000^*^ | .846937 | .019 | .52277 | 5.45723 |
|  |  |  | 2 | -.085000 | .831152 | .999 | -2.49847 | 2.32847 |
| *.P < 0.05。 | | | | | | | | |

**Homogeneous Subsets**

| **Acetic** | | | | |
| --- | --- | --- | --- | --- |
|  | GROUP | N | alpha = 0.05 | |
|  |  |  | 1 | 2 |
| Duncan^a^ | 1 | 6 | 3.53667 |  |
|  | 3 | 6 |  | 4.90833 |
|  | 2 | 6 |  | 5.21667 |
|  | Sig. |  | 1.000 | .485 |
| Means for groups in homogeneous subsets are displayed. | | | | |
| Uses Harmonic Mean Sample Size = 6.000. | | | | |

| **Propionic** | | | | |
| --- | --- | --- | --- | --- |
|  | GROUP | N | alpha = 0.05 | |
|  |  |  | 1 | 2 |
| Duncan^a^ | 1 | 6 | 3.58833 |  |
|  | 2 | 6 |  | 4.37500 |
|  | 3 | 6 |  | 4.59667 |
|  | Sig. |  | 1.000 | .534 |
| Means for groups in homogeneous subsets are displayed. | | | | |
| Uses Harmonic Mean Sample Size = 6.000. | | | | |

| **Butyric** | | | | |
| --- | --- | --- | --- | --- |
|  | GROUP | N | alpha = 0.05 | |
|  |  |  | 1 | 2 |
| Duncan^a^ | 1 | 6 | .78833 |  |
|  | 2 | 6 |  | 1.35167 |
|  | 3 | 6 |  | 1.52833 |
|  | Sig. |  | 1.000 | .436 |
| Means for groups in homogeneous subsets are displayed. | | | | |
| Uses Harmonic Mean Sample Size = 6.000. | | | | |

| **Isovaleric** | | | |
| --- | --- | --- | --- |
|  | GROUP | N | alpha = 0.05 |
|  |  |  | 1 |
| Duncan^a^ | 1 | 6 | .21500 |
|  | 2 | 6 | .24833 |
|  | 3 | 6 | .30667 |
|  | Sig. |  | .077 |
| Means for groups in homogeneous subsets are displayed. | | | |
| Uses Harmonic Mean Sample Size = 6.000. | | | |

| **Valeric** | | | | |
| --- | --- | --- | --- | --- |
|  | GROUP | N | alpha = 0.05 | |
|  |  |  | 1 | 2 |
| Duncan^a^ | 3 | 6 | .04333 |  |
|  | 1 | 6 |  | .26500 |
|  | 2 | 6 |  | .27667 |
|  | Sig. |  | 1.000 | .910 |
| Means for groups in homogeneous subsets are displayed. | | | | |
| Uses Harmonic Mean Sample Size = 6.000. | | | | |

| **Total** | | | | |
| --- | --- | --- | --- | --- |
|  | GROUP | N | alpha = 0.05 | |
|  |  |  | 1 | 2 |
| Duncan^a^ | 1 | 6 | 8.39333 |  |
|  | 3 | 6 |  | 11.38333 |
|  | 2 | 6 |  | 11.46833 |
|  | Sig. |  | 1.000 | .925 |
| Means for groups in homogeneous subsets are displayed. | | | | |
| Uses Harmonic Mean Sample Size = 6.000. | | | | |
