# Supplementary material for: The influence of food processing methods on serum parameters, apparent total-tract macronutrient digestibility, fecal microbiota and SCFA content in adult beagles
Source: PLoS One. 2022 Jan 19;17(1):e0262284. doi: 10.1371/journal.pone.0262284 (PMC8769318; doi:10.1371/journal.pone.0262284)
Supplement: Attachment — Submitted filename: Response to Reviewers0819.docx [file pone.0262284.s009.docx]

Dear Dr. Chaves,

Thank you very much for carefully check with our manuscript “The influence of food processing methods on serum parameters, apparent total-tract macronutrient digestibility, fecal microbiota and SCFA content in adult beagles”.

This manuscript was revised according to the reviewer/editor comments, and the revised sentences were marked in red color. The reply to comments is listed in “revision checklist” (in red color).

Kind regards,

Dr. Xuan Cai

Revision checklist

1. Manuscript is not formatted for Plos One.

Re: Thank you for your careful check. We have modified the format of this manuscript according to the instructions provided in the website. Although we have tried our best to modify the format of the manuscript, we believe that there are still some format problems in the manuscript. Please point it out in the next revision, and we will correct it seriously.

2. Provide specific number of each of the AOAC methods.

Re: Thank you for your careful check. This has been corrected (line 86-88).

3. Statistical analysis is poor described. Authors must breakdown the statistical analysis for the animal experiment and amplicon sequencing.

Re: Thank you for your careful check. We have rewritten the "statistical analysis" and I don't know if it meets the requirements of the magazine. The data processing in the part of "amplicon sequencing" is described in a section in "Materials and Methods", so the description was not distinguished.

4. For the animal experiment, what were the random and fixed effected used in the model? What was the experimental unit? Were repeated measure analysis used? What is the power for the number of animals used? Why Duncan?

Re: Thank you for your advice. I'm sorry I think we may not fully understand what you mean. I guess your suggestion is for the use of SAS as statistical software for analysis. We are not familiar with SAS software. In this study (except for PCA analysis of sequencing data), all comparisons between groups are based on SPSS software. We did not mention the concept of "random and fixed effected used in the model" in our previous analysis. Correspondingly, we guessed and answered based on the literal meaning. In this study, the health status of the dogs, the raw materials and ingredients of the feed are fixed, and the only variable is the processing method of the feed.

In this study, one dog is a repeat. Therefore, the number of repetitions in this study is six. The supplementary description was added to line 101 and table notes to allow readers to more clearly define the experimental design.

As for why Duncan analysis is used. Well, it seems that there is no absolute standard for which hypothesis testing method to choose. We are based on the results of a Chinese degree thesis (Evalluation of multiple comparison methods with quantitative data): "When the first aim is to explore the difference between the neans of each groups, the LSD and Duncan methods are first selected..." At the same time, the SPPS software expresses the results of Duncan analysis more clearly. Many hypothesis tests in the literature with similar experimental design also use Duncan analysis (Liu et al., 2014. doi: 10.1371/journal.pone.0106412; Song et al., doi: 2017. 10.3382/ps/pex163), so we use Duncan analysis for comparison.

5. Tables 3 to 5: please provide the P-values Type 3 Tests of Fixed Effects. Defined HTS: Tables must stand alone so any abbreviation must be defined regardless if previously defined.

Re: Thank you for your careful check. These have been corrected (Tables 2 to 7, Fig 1 & Fig 2).

6. LSMEANS of Erysipelatoclostridium zero? It should be either not detected or increase the decimals.

Re: Thank you for your careful check. These have been corrected (Line 296).

7. Please include captions for your Supporting Information files at the end of your manuscript, and update any in-text citations to match accordingly. Please see our Supporting Information guidelines for more information: http://journals.plos.org/plosone/s/supporting-information.

Re: Thank you for your careful check. These have been corrected.

**Journal Requirements:**

1. Please ensure that your manuscript meets PLOS ONE's style requirements, including those for file naming. The PLOS ONE style templates can be found at

https://journals.plos.org/plosone/s/file?id=wjVg/PLOSOne_formatting_sample_main_body.pdf and https://journals.plos.org/plosone/s/file?id=ba62/PLOSOne_formatting_sample_title_authors_affiliations.pdf

Re: Thank you for your careful check. We have modified the format of this manuscript according to the instructions provided in the website. Although we have tried our best to modify the format of the manuscript, we believe that there are still some format problems in the manuscript. Please point it out in the next revision, and we will correct it seriously.

2. To comply with PLOS ONE submissions requirements, in your Methods section, please provide additional information regarding the experiments involving animals and ensure you have included details on (1)housing condition, (2) methods of sacrifice, (3) amount of blood taken from each animal (4) where the blood was taken and (5) methods of anaesthesia if used for blood collection.

Re: Thank you for your careful check. (1) Housing condition was described in Line 105-108; (2) No animal sacrificed in this study; (3) Amount of blood taken from each animal was described in Line 113; (4) The place blood was taken described in Line 114; (5) Anaesthesia was not used in this study.

3. We note that you have indicated that data from this study are available upon request. PLOS only allows data to be available upon request if there are legal or ethical restrictions on sharing data publicly. For more information on unacceptable data access restrictions, please see http://journals.plos.org/plosone/s/data-availability#loc-unacceptable-data-access-restrictions.

Re: Thank you for your advice. Raw sequences reads for this study can be found in the NCBI sequence read archive BioProject ID: PRJNA733866. Other relevant data are within the paper and its Supporting Information files.

E-mail for PONE-D-21-20446R1:

1. Please ensure that you refer to Table 2 in your text as, if accepted, production will need this reference to link the reader to the Table.

Re: Thank you for your advice. It has been added in line 88.
