# Supplementary material for: The influence of food processing methods on serum parameters, apparent total-tract macronutrient digestibility, fecal microbiota and SCFA content in adult beagles
Source: PLoS One. 2022 Jan 19;17(1):e0262284. doi: 10.1371/journal.pone.0262284 (PMC8769318; doi:10.1371/journal.pone.0262284)
Supplement: Attachment — Submitted filename: Response to Reviewers_R2.docx [file pone.0262284.s010.docx]

Dear Dr. Chaves,

Thank you very much for carefully check with our manuscript “The influence of food processing methods on serum parameters, apparent total-tract macronutrient digestibility, fecal microbiota and SCFA content in adult beagles”.

This manuscript was revised according to the reviewer/editor comments, and the revised sentences were marked in red color. The language of the manuscript has been edited by a company named “International Science Editing”. The reply to comments is listed in “revision checklist” (in red color).

Kind regards,

Dr. Xuan Cai

Revision checklist

1. This paper requires a review by a professional editor in order to correct the grammatical, spelling, and punctuation errors. I began to fix some of the mistakes, but they were too numerous to continue. Some of these errors make it difficult to understand the sentences, especially in the materials and methods, and results sections. Therefore, I strongly recommend a professional editor.

Re: Thank you for your careful check. We have carefully checked the manuscript and the language of this manuscript has been edited by a company named “International Science Editing” (https://www.internationalscienceediting.com/).

2. L179: Please provide a brief description validating the use of one-way ANOVA. Ex.: does the data follow parametric assumptions, validated by a KS test (normality) and Levene’s test (homogeneity of variance)?

Re: Thank you for your advice. This has been corrected (Line 183-186).

3. Table 4: The significance letters are not labeled consistently. Specifically, refer to item “TG” under HTS

Re: Thank you for your careful check. It has been corrected (Table 4).

4. Table 6: In addition to the p-values, please add significance letters to keep it consistent with the other tables.

Re: Thank you for your careful check. It has been corrected (Table 6, Line 303).

5. L407-414: Written exactly as in the abstract. Please re-word this.

Re: Thank you for your careful check. It has been corrected (Line 412-414)..

6. Figure 1c: It would be better to start the y-axis numbering from 0% instead of 80%, and then include a break in the bar graphs instead.

Re: Thank you for your advice. These have been corrected.

7. The authors have set about testing the question whether there is any nutritional difference between feeding a diet that has been processed in 3 different ways: raw, pasteurised or high temperature sterilization. The premise underlying this project is the statement in the first line of the manuscript: “Food processing methods have a huge influence on the health of dogs.” The experimental design and the methods of analysis are all appropriate for determining any biological effects of feeding to a group of beagle dogs, the same diet processed in 3 different ways. A wide range of assays were undertaken, and statistically different mean values were found in protein digestibility, serum triglycerides, platelet and neutrophil populations in serum and the species range and population proportion of faecal microorganisms of dogs fed on these differently processed diets.

The experimental approach and its technical execution were entirely appropriate to test the hypothesis that the 3 methods of food preparation would show differences in various biological features of the dogs being studied. However, how do the authors justify the findings in terms of the opening sentence of their manuscript: “Food processing methods have a huge influence on the health of dogs.”? What is the evidence that there was any difference between the processing methods and the health of the dogs being studied? Certainly, statistical differences were found in some of the responses being measured, but these differences were so small that it is difficult to see how they would have had any impact on the “health of the dogs”. It is not surprising that heat treatment would affect digestibility of protein. It probably also would have affected starch digestibility in the rice component of the food formulation. Hence the composition of the digestion residue being presented to the microbiome in the large intestine would be different between the 3 processing methods. This would be the likely explanation for the findings of this study. This conclusion should have been pointed out in the Discussion section of the paper, along with an assessment that all of the significantly different changes, observed between the 3 processing methods, would have had negligible effects on the “health” of the dogs. The study was worth doing, but the results are entirely unsurprising.

Re: Thank you very much for your pertinent evaluation. Health is a difficult concept to define, and different researchers may think differently. Because dogs and cats must be sampled non-destructively, the observable indicators are usually the measurement indicators of serum, feces, and urine, which are usually measured by researchers during the research process, such as PMID: 29893876, 30110340, 33480132. But this article began to define it as health is indeed not rigorous enough, so we have revised it accordingly.

The prosperity of the pet industry in recent years has brought many novel processing techniques, but the research on the impact of these processing techniques on the health of dogs and cats is still very limited. There have been some studies in the past, but due to processing difficulties, many researchers use different comparing formulas of dry food and wet food, although there are many interesting conclusions, it is difficult to rule out the interference of different formulas and raw materials. This study is trying to use the same formula and the same raw material to process dog food with different characteristics, and analyze its influence on some physiological and biochemical indicators of beagle dogs, and supplement and verify previous studies. In this sense, we think it can help promote the development of the pet food industry.

However, this study does have some problems. Because of the "schedule" problem of experimental animals, we chose a group test. This resulted in a small number of beagle dogs in each group and large differences within the group, so it is difficult to perform in many analyses significant difference. If we have the opportunity to continue to engage in relevant research, we will be more inclined to use a larger sample size staged trial rather than grouped trials.

**Journal Requirements:**

1. Please ensure that your manuscript meets PLOS ONE's style requirements, including those for file naming. The PLOS ONE style templates can be found at

https://journals.plos.org/plosone/s/file?id=wjVg/PLOSOne_formatting_sample_main_body.pdf and https://journals.plos.org/plosone/s/file?id=ba62/PLOSOne_formatting_sample_title_authors_affiliations.pdf

Re: Thank you for your careful check. We have modified the format of this manuscript according to the instructions provided in the website. Although we have tried our best to modify the format of the manuscript, we believe that there are still some format problems in the manuscript. Please point it out in the next revision, and we will correct it seriously.

2. To comply with PLOS ONE submissions requirements, in your Methods section, please provide additional information regarding the experiments involving animals and ensure you have included details on (1)housing condition, (2) methods of sacrifice, (3) amount of blood taken from each animal (4) where the blood was taken and (5) methods of anaesthesia if used for blood collection.

Re: Thank you for your careful check. (1) Housing condition was described in Line 106-109; (2) No animal sacrificed in this study; (3) Amount of blood taken from each animal was described in Line 113; (4) The place blood was taken described in Line 114-115; (5) Anaesthesia was not used in this study.

3. We note that you have indicated that data from this study are available upon request. PLOS only allows data to be available upon request if there are legal or ethical restrictions on sharing data publicly. For more information on unacceptable data access restrictions, please see http://journals.plos.org/plosone/s/data-availability#loc-unacceptable-data-access-restrictions.

Re: Thank you for your advice. Raw sequences reads for this study can be found in the NCBI sequence read archive BioProject ID: PRJNA733866. Other relevant data are within the paper and its Supporting Information files.
