# Supplementary material for: The influence of food processing methods on serum parameters, apparent total-tract macronutrient digestibility, fecal microbiota and SCFA content in adult beagles
Source: PLoS One. 2022 Jan 19;17(1):e0262284. doi: 10.1371/journal.pone.0262284 (PMC8769318; doi:10.1371/journal.pone.0262284)
Supplement: Attachment — Submitted filename: Response to Reviewers_R3.docx [file pone.0262284.s011.docx]

Dear Dr. Chaves,

Thank you very much for carefully check with our manuscript “The influence of food processing methods on serum parameters, apparent total-tract macronutrient digestibility, fecal microbiota and SCFA content in adult beagles”.

This manuscript was revised according to the reviewer comments, and the revised sentences were marked in red color.

Kind regards,

Dr. Xuan Cai

Revision checklist

1. The authors have satisfactorily changed the wording of their manuscript to avoid implying that the state of health of the dogs was affected by the method of food processing. Certainly some stattstically significant differences were found in some of the measurements being made. These differences were small and would not indicate any change in the state of health of the dogs.

There is one minor correction that is needed. In Tables 6 and 7, mean values plus and minus standard errors are given. The units for these values should be stated, as has been done for the data in the other tables.

Re: Thank you for your careful check. We corrected this error in Tables 6 and7 and added units.
