# Supplementary material for: The influence of food processing methods on serum parameters, apparent total-tract macronutrient digestibility, fecal microbiota and SCFA content in adult beagles
Source: PLoS One. 2022 Jan 19;17(1):e0262284. doi: 10.1371/journal.pone.0262284 (PMC8769318; doi:10.1371/journal.pone.0262284)
Supplement: Attachment — Submitted filename: Response to Reviewers_R4.docx [file pone.0262284.s012.docx]

Dear Dr. Chaves,

Thank you very much for carefully check with our manuscript “The influence of food processing methods on serum parameters, apparent total-tract macronutrient digestibility, fecal microbiota and SCFA content in adult beagles”.

Because this modification is for the supplementary file table, the text has not been modified, so there is no 'Revised Manuscript with Track Changes' file for this modification.

Kind regards,

Dr. Xuan Cai

Revision checklist

1. Did the authors run stats on Table S1? if so please present the P-values.

Re: Thank you for your careful check. We have added a column with P-values.

2. I just noticed that in Table S2, authors mentioned "Significant (P < 0.05) differences among Duncan analyze in the same row are indicated by different superscript letters." but there are no letters on the LSMEANS. Can you please also add a column with P-values?

Re: Thank you for your careful check. The sentence "Significant (P < 0.05) differences among Duncan analyze in the same row are indicated by different superscript letters." Should deleted here for no significant (P < 0.05) difference existed for the data in this table. We have added a column with P-values in Table S2, and also Table S3.

3. S1, S2 & S3 files must be written in English not in Chinese.

Re: Thank you for your careful check. We have revised these.
